# Prognostic and diagnostic validity of p16/Ki-67, HPV E6/E7 mRNA, and HPV DNA in women with ASCUS: a follow-up study

**DOI:** 10.1186/s12985-019-1251-4

**Published:** 2019-11-21

**Authors:** Chenchen Ren, Yuanhang Zhu, Li Yang, Xiaoan Zhang, Ling Liu, Zhaoxin Wang, Dongyuan Jiang

**Affiliations:** 1 Department of Obstetrics and Gynecology, The Third Affiliated Hospital of Zhengzhou University, No. 7, Front Kangfu Street, Zhengzhou, 450052 Henan Province People’s Republic of China; 2 Department of Imaging, The Third Affiliated Hospital of Zhengzhou University, Zhengzhou, 450052 People’s Republic of China

**Keywords:** HPV, E6/E7, mRNA, p16/Ki-67, ASCUS, Follow-up

## Abstract

**Background:**

We evaluated the prognostic and diagnostic ability of p16/Ki-67 immunocytochemistry, HPV E6/E7 mRNA testing and HPV DNA assay in triaging ASCUS to find a way to manage cervical lesions more effectively.

**Methods:**

We conducted a prospective study through follow-up. The detection methods of the three factors: p16/Ki-67 immunocytochemistry conducted by using the CINtec® Plus Kit, E6/E7 mRNA testing by QuantiVirus®HPV E6/E7 mRNA assay and DNA by Hybrid Capture 2 assay.

**Results:**

One hundred three women with ASCUS satisfied requirements and completed the entire follow-up process. All CIN2+ occurred in women who were mRNA positive at baseline, none in mRNA negative. 100% (6/6) patients with CIN2+ were HPV DNA assay positive, 100% (6/6) were HPV E6/E7 mRNA testing positive and 50.0% (3/6) were p16/Ki-67 immunocytochemistry positive. The risk ratio of E6/E7 mRNA test was 57.306 (95% CI 0.077–42,400.545). For endpoint of CIN2+, the sensitivity between HPV DNA assay and HPV E6/E7 mRNA testing is no statistical difference, but statistical difference exists between HPV E6/E7 mRNA testing vs. p16/Ki-67 immunocytochemistry (*χ*^2^ = 5.718, *P* = 0.023) and HPV DNA assay vs. p16/Ki-67 immunocytochemistry (*χ*^2^ = 5.718, *P* = 0.023). The specificity of E6/E7 mRNA testing, p16/Ki-67 and DNA assay in triaging ASCUS was 44.33, 75.26 and 11.34% respectively and is all statistical difference (*χ*^2^ = 26.277, *P* < 0.001(HPV DNA assay vs. HPV E6/E7 mRNA testing), *χ*^2^ = 19.297, *P* < 0.001(HPV E6/E7 mRNA testing vs. p16/Ki-67 immunocytochemistry), *χ*^2^ = 80.707, *P* < 0.001(HPV DNA assay vs. p16/Ki-67 immunocytochemistry). The expression level of 2097.09 copies/ml was the optimal cut-off value for HPV E6/E7 mRNA testing to diagnose CIN2+, the sensitivity and specificity was 61.1 and 68.2%.

**Conclusions:**

High expression of HPV E6/E7 mRNA could be a good candidate as a diagnostic biomarker to triage ASCUS superseding HPV DNA. p16/Ki-67 immunocytochemistry is suggested to be a good tool to triage ASCUS, but it reduced the sensitivity of diagnosis when improves the diagnostic specificity.

## Introduction

The aim in primary screening for cervical cancer is to find out the high-grade cervical intraepithelial lesions and lesions which may develop into high-grade cervical intraepithelial lesions in the future. Liquid-based cervical cytology acted as the most important strategy for cervical cancer screening have reduced cervical cancer incidence and mortality during the last decades [1]. According to the Bethesda System 2001 guidelines [2], atypical squamous cells of undetermined significance (ASCUS) is diagnosed when the characteristics of cells are significantly more than inflammatory cell changes, but the number and quality were not enough to diagnose epithelial malignant lesions. According to the statistics, 59.3% of abnormal Papanicolaou (Pap) smear result is ASCUS [3]. Unfortunately, ASCUS is an ambiguous cytology result. The histological results of ASCUS contain normal cervical tissue, intraepithelial neoplasia and cervical cancer, and the risk of cervical high-grade intraepithelial neoplasia is only 9.7% [4, 5]. HPV DNA testing is a useful method in the cervical cancer screening among women over 30 years old [6, 7], and became a triage test in ASCUS for many years [8]. HR-HPV DNA testing is a good triage option but neoplastic transformation is a rare complication of HPV infection and many of HPV infection are mostly transient [9]. There is study found that HPV DNA assay can’t find high-grade intraepithelial neoplasia in highly specificity [4].

Biomarkers of cervical cancer are needed to identify those HPV infections which may develop into a high-grade lesion. The most important process in cervical carcinogenesis progression is that HPV insert its sequence, especially the abnormal expression of E6 and E7 oncogenes, into the host genome. p16INK4a (p16) -overexpression is thought as a sign for deregulating E7 expression [10, 11]. Ki-67 is a cell proliferation marker [12, 13]. Under physiological conditions, The expressions of p16 and Ki-67 do not occur simultaneously [14]. New biomarker assays predicting the post-transcriptional status of HPV may improve the accuracy of cervical cancer screening [15].

HPV E6/E7 mRNA test and p16/Ki-67 immunocytochemistry has been proposed as promising biomarkers for HPV oncogene expression. Previous studies had showed that HPV E6/E7 mRNA test and p16/Ki-67 immunocytochemistry have a large clinical value in cervical cancer screening [16–18], however, whether they can be a useful method in triaging ASCUS has not been determined. Here, we evaluated the prognostic and diagnostic ability of p16/Ki-67 immunocytochemistry, HPV E6/E7 mRNA testing and HPV DNA assay to identify women with ASCUS who will develop high-grade intraepithelial neoplasia during follow up by a cross-sectional study.

## Methods

### Study population and study design

The study population consisted of female who visited outpatient clinic of gynecology department of the third affiliated hospital of Zhengzhou University from September 2015 to September 2017. Follow up was performed according to local colposcopy protocols. 300 women with ASCUS entered our study and all of them underwent Pap test, colposcopy biopsy and histopathological examination. These patients with ASCUS and histological diagnosis of cervical inflammation or mild cervical intraepithelial neoplasia (grade 1 cervical intraepithelial neoplasia, CIN1) and no cervical destructive treatment or local and/or systemic medical treatment were recruited in this follow-up study. During the follow-up, 54 cases were excluded because the histological result is CIN2, CIN3 or cervical cancer. 28 cases were excluded due to cervical destructive treatment, 18 cases were excluded because positive cytology or HPV was found but not followed by colposcopy, 81 cases were excluded due to local and/or systemic medical treatment, 16 cases were excluded due to loss of follow-up. At last, 103 cases satisfied the requirements and completed the entire follow-up process. All subjects were followed up until 1 September 2018 and the duration of follow-up is at least 12 months (12 months–24 months). one or more cervical cancer screening was carried out and the last follow up episode determined the final follow-up result. When the last follow up episode is a negative cytology and HPV DNA, the final follow-up result defined as negative. When the last follow up episode is a positive cytology or HPV DNA, the final follow-up result was defined by colposcopy and histopathology. For women with cervical inflammation or CIN1, the final follow-up result of CIN2 or more serious defined as progression of cervical lesion. This study was reviewed and approved by the Ethics Committee of The Third Affiliated Hospital of Zhengzhou University. All women signed the consent forms and were informed regarding the purpose of the proposed study.

### Liquid based cytology

The technology of LBC was used for the cytology test. Thinlayer slides were prepared using the Thin Prep 2000 Processor (Cytyc Corporation, Marlborough, MA, USA) according to the manufacturer’s instructions. The cytological specimens were reported using the 2001 Bethesda Reporting System Criteria [2].

### p16/Ki-67 immunocytochemistry

A second cytology slide was prepared from the residual LBC specimen using a T2000 slide processor (Hologic, Bedford, MA, USA). Immunostaining of cervical cytology slides for p16/Ki-67 was performed using the CINtec® Plus Kit (Roche mtm laboratories AG, Heidelberg, Germany) according to the manufacturer’s instructions. A case was considered positive if one or more cervical epithelial cell(s) stained both with a brown cytoplasmic stain (p16) and a red nuclear (Ki-67) irrespective of the interpretation of morphologic abnormalities. Slides without any double-stained cells were called negative for p16/Ki-67 dual-stain cytology [19].

### HPV E6/E7 mRNA testing

Residual LBC samples were also used for the detection of E6/E7 mRNA of 14 HR-HPV types by QuantiVirus®HPV E6/E7 mRNA assay (Kodia, Henan, China) according to the manufacturer’s instructions. If the copy number was greater than or equal to 1.0, the QuantiVirus® HPV assay result for the patient was positive. Otherwise, the result was negative [20].

### HPV DNA assay

HPV DNA was detected by Hybrid Capture 2 assay (HC2, Digene, Gaithersburg, MD, USA), according to the manufacturer’s instructions. RLU/CO ≥1 was identified as positive.

### Colposcopy and histological diagnosis

Women with ASCUS Pap smears underwent colposcopy biopsy within 4 weeks after cytology test, according to colposcopy operating norms. All histological slides were diagnosed according to current World Health Organization classification. In this study, the diagnoses of CIN2, CIN3 and carcinoma are referred as ≥ CIN2 (CIN2 +). Histology was regarded as the “gold standard”, but for those whose cytology and HPV DNA were negative, the final results of follow-up were considered to be negative. The diagnoses of cytology and HPV DNA negative (no biopsy), cervical inflammation and CIN1 are referred as < CIN1- (CIN1-).

### Statistics

Statistical analysis was performed using SPSS 21.0 software. Chi-square tests were used to compare differences between groups when type of date was qualitative variable. Youden’s index was calculated as sensitivity% + specificity% - 100. Receiver operating characteristic (ROC) curve was used to assess the optimal diagnosis of E6/E7 mRNA expression testing. The risk of CIN2+ in ASCUS was evaluated by Cox’s proportional hazards regression model. All tests were two-sided and *p* value < 0.05 was considered the cut-off level for statistical significance for all analysis.

## Results

### The follow-up result of women with ASCUS pap smear

One hundred three women with ASCUS satisfied requirements and completed the entire follow-up process. Table 1 shows the baseline characteristics of the 103 subjects. Mean follow-up time was 15.3 months, ranging from 12 to 24 months. During follow up, only 6 high-grade CIN, five CIN2, and one CIN3 were identified. The risk ratio was 5.598 (95% CI 0.652–48.073) in the whole cohort. All six CIN2+ occurred in women who were mRNA positive at baseline, none in women with mRNA negative at baseline. The risk ratio of HPV E6/E7 mRNA test was 57.306 (95% CI 0.077–42,400.545). The risk ratio of HPV DNA testing and p16/Ki-67 immunocytochemistry was also calculated (Table 1).

**Table 1:** Follow up histology results for women with ASCUS by different test results at baseline

Follow-up result	different test results at baseline							
	Histological Results		DNA		E6/E7 mRNA		p16/Ki-67	
	Inflammation	CIN1	Negative	Positive	Negative	Positive	Negative	Positive
No biopsy	33	29	11	51	33	29	52	10
Inflammation	9	8	0	17	5	12	16	1
CIN1	7	11	0	18	5	13	5	13
CIN2	0	5	0	5	0	5	2	3
CIN3	1	0	0	1	0	1	1	0
Risk ratio	5.598		23.842		57.306		3.629	
95%CI	0.652–48.073		0.000–2,213,315.430		0.077–42,400.545		0.726–18.131	

*CIN* cervical intraepithelial neoplasia, *DNA* HPV DNA assay, *E6/E7 mRNA* HPV E6/E7 mRNA testing, *p16/Ki-67* p16/Ki-67 immunocytochemistry

### Test positivity in relation to the histopathologic diagnosis

We calculated the positive rate of different methods in different levels of cervical tissue. The overall test positivity was 89.3% (92/103) in HPV DNA assay, 58.3% (60/103) in HPV E6/E7 mRNA testing and 26.2% (27/103) in p16/Ki-67 immunocytochemistry. HPV E6/E7 mRNA testing and p16/Ki-67 immunocytochemistry was less frequently positive in ASCUS than HPV DNA test. 82.3% (51/62) patients with no biopsy were HPV DNA assay positive, 46.8% (29/62) were HPV E6/E7mRNA testing positive and 16.1% (10/62) were p16/Ki-67 immunocytochemistry positive. 100% (17/17) patients with inflammation were HPV DNA assay positive, 70.6% (12/17) were HPV E6/E7 mRNA testing positive and 5.9% (1/17) were p16/Ki-67 immunocytochemistry positive. 100% (18/18) patients with CIN1 were HPV DNA assay positive, 72.2% (13/18) were HPV E6/E7 mRNA testing positive and 72.2% (13/18) were p16/Ki-67 immunocytochemistry positive. 100% (6/6) patients with CIN2+ were HPV DNA assay positive, 100% (6/6) were HPV E6/E7 mRNA testing positive and 50.0% (3/6) were p16/Ki-67 immunocytochemistry positive. The positive rates of HPV DNA assay, HPV E6/E7mRNA testing and p16/Ki-67 immunocytochemistry in CIN1- and CIN2+ were statistically compared in Table 2.

**Table 2:** Positive rate of HPV DNA assay, HPV E6/E7mRNA testing and p16/Ki-67 immunocytochemistry in CIN1- and CIN2+

Test	positive rate		*χ*^2^ value	*p* value
	CIN1-	CIN2+		
DNA	88.7% (86/97)	100% (6/6)	0.037	0.848
E6/E7mRNA	55.7% (54/97)	100% (6/6)	6.749	0.009
p16/Ki-67	17.5% (24/97)	50.0% (3/6)	1.649	0.199

CIN1-: The diagnoses of cytology and HPV DNA negative (no biopsy), cervical inflammation and CIN1 are referred as < CIN1(CIN1 -); CIN2+: the diagnoses of CIN2, CIN3 and carcinoma are referred as ≥ CIN2 (CIN2 +). DNA: HPV DNA assay; E6/E7 mRNA: HPV E6/E7 mRNA testing; p16/Ki-67: p16/Ki-67 immunocytochemistry

### Comparison of the expression level of HPV E6/E7 mRNA testing in different cervical lesions

**Fig. 1:**
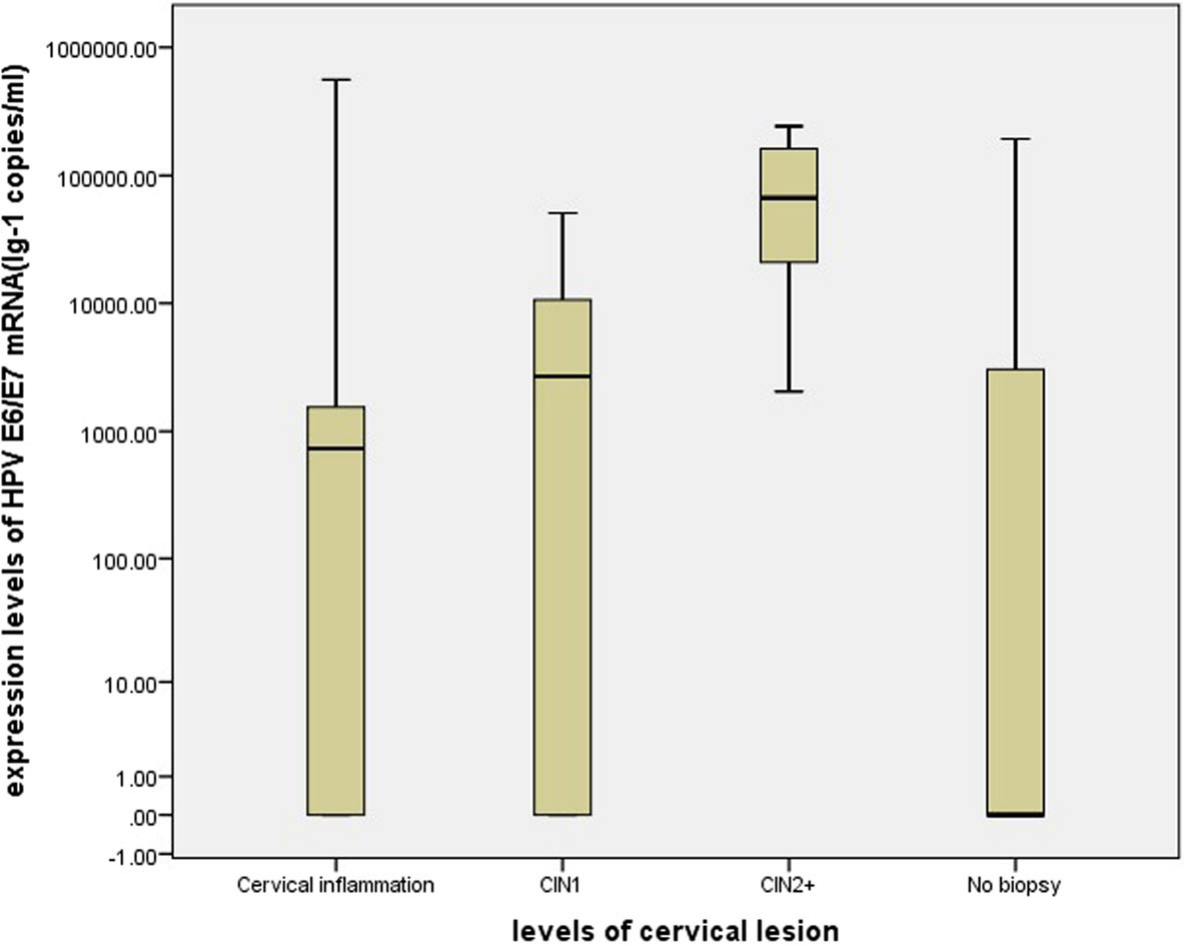
The expression level of HR-HPV E6/E7 mRNA in different levels of cervical lesion. Note: CIN 1: grade 1 cervical intraepithelial neoplasia; CIN2+: the diagnoses of CIN2, CIN3 and carcinoma are referred as ≥ CIN2

The expression level of HPV E6/E7 mRNA testing in different cervical lesions is shown in Fig. 1 and Table 3. The E6/E7 mRNA showed a higher expression levels in CIN2+ than in CIN1−, and the differences between the lesions were statistically significant (*P* < 0.05).

**Table 3:** The results of HPV E6/E7 mRNA expression levels in different levels of cervical lesion

Group	expression levels (copies / ml)^a^	Average rank	*Z* value	*p* value
No biopsy	0(0–3254.10)			
Inflammation	733.44(0–2222.60)			
CIN1	2738.75(0–10,702.87)			
CIN2+	94,116.51 (16,207.12–182,555.36)			
CIN1-	522.45(0–3841.54)	49.60	−3.407	0.001
CIN2+	94,116.51 (16,207.12–182,555.36)	90.83		

^a^ Median of mRNA load, with 25th–75th percentile in parentheses; CIN: cervical intraepithelial neoplasia; CIN1-: The diagnoses of cytology and HPV DNA negative (no biopsy), cervical inflammation and CIN1 are referred as < CIN1(CIN1 -); CIN2+: the diagnoses of CIN2, CIN3 and carcinoma are referred as ≥ CIN2 (CIN2 +)

### Diagnostic sensitivity and specificity of the different tests

**Fig. 2:**
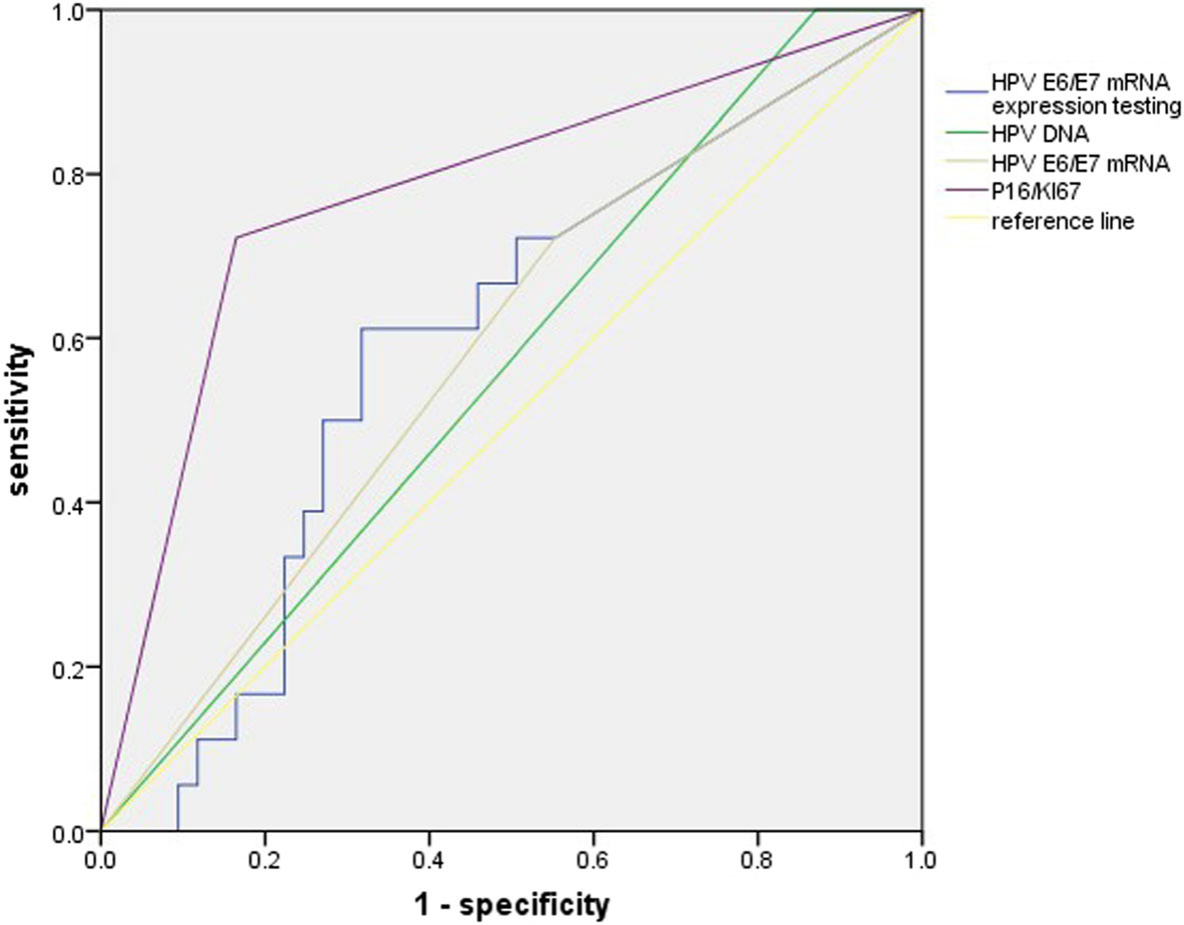
ROC curve of HPV DNA assay, HPV E6/E7 mRNA testing and p16/Ki-67 immunocytochemistry for detecting CIN2+

The sensitivity, specificity, PPV, NPV and Youden index for CIN2+ of the HPV DNA assay, HPV E6/E7 mRNA testing and p16/Ki-67 immunocytochemistry were shown in Table 4. For endpoint of CIN2+, the sensitivity between HPV DNA assay and HPV E6/E7 mRNA testing is no statistical difference (all sensitivities are 100, 95%CI: 60.97, 100), but statistical difference exists between HPV E6/E7 mRNA testing vs. p16/Ki-67 immunocytochemistry (*χ*^2^ = 5.718, *P* = 0.023) and HPV DNA assay vs. p16/Ki-67 immunocytochemistry (*χ*^2^ = 5.718, *P* = 0.023). The specificity is all statistical difference (*χ*^2^ = 26.277, *P* < 0.001(HPV DNA assay vs. HPV E6/E7 mRNA testing), *χ*^2^ = 19.297, *P* < 0.001(HPV E6/E7 mRNA testing vs. p16/Ki-67 immunocytochemistry), *χ*^2^ = 80.707, *P* < 0.001(HPV DNA assay vs. p16/Ki-67 immunocytochemistry). ROC curve was used to determine an optimal cut-off value and further demonstrate the diagnostic performance of HPV E6/E7 mRNA testing for detecting CIN2+ (Fig. 2). The expression level of 2097.09 copies/ml was the optimal cut-off value for HPV E6/E7 mRNA testing to diagnose CIN2+, and at this time, the sensitivity and specificity was 61.1 and 68.2%. The accuracy of different assay was also displayed in ROC curve by calculating the area under the curve (AUC). Among the three tests, the AUC of p16/Ki-67 immunocytochemistry was the largest (Table 5).

**Table 4:** Sensitivity, specificity, PPV, NPV and Youden index of HPV DNA assay, HPV E6/E7mRNA testing and p16/Ki-67 immunocytochemistry

Test	Sensitivity	Specificity	PPV	NPV	Youden index
	(95% CI)	(95% CI)	(95% CI)	(95% CI)	
DNA	100%	11.34%	6.52%	100%	11.34
	(60.97, 100)	(6.45, 19.71)	(3.02, 13.51)	(74.12, 100)	
E6/E7 mRNA	100%	44.33%	10.00%	100%	44.33
	(60.97, 100)	(34.85, 54.24)	(4.67, 20.15)	(91.80, 100)	
p16/Ki67	50%	75.26%	11.11%	96.05%	25.26
	(18.76, 81.24)	(65.82, 82.77)	(3.85, 28.06)	(89.03, 98.65)	

*PPV* positive predictive value, *NPV* negative predictive value. Results in % with 95% confidence interval (95% CI). DNA: HPV DNA assay; E6/E7 mRNA: HPV E6/E7 mRNA testing; p16/Ki-67: p16/Ki-67 immunocytochemistry

**Table 5:** The area under ROC curve of HPV DNA assay, HPV E6/E7mRNA testing, HPV E6/E7mRNA and p16/Ki-67 immunocytochemistry

Test	AUC	95% CI
HPV DNA	0.565	(0.430–0.700)
HPV E6/E7 mRNA	0.585	(0.444–0.725)
HPV E6/E7 mRNA expression	0.593	(0.456–0.731)
p16/Ki67	0.779	(0.649–0.908)

*AUC* the area under the curve. Results in % with 95% confidence interval (95% CI). HPV E6/E7 mRNA expression means that 2097.09 copies/ml act as cut-off value to judge negative and positive of HPV E6/E7 mRNA testing and calculate the AUC

## Discussion

Although the association between E6/E7 mRNA, p16/Ki-67 overexpression and high grade CIN of ASCUS has been demonstrated in some studies [18, 21], our study is one of the first follow up studies for ASCUS with negative or CIN 1 results. Our results suggest that both p16/Ki-67 immunocytochemistry and HPV E6/E7 mRNA testing may have a prognostic and diagnostic value for cervical precancer, even if the small number of CIN2+ detected and chance might influence our results. It is undeniable that the effectiveness of cervical cancer screening is closely related to the ASCUS diagnostic rate, resulting in a lower number of ASCUS patients. This also resulted in small number of participants included in our experiment. Furthermore, because ASCUS is not a particularly serious pathology in clinic, our follow-up time is not very long in order to ensure a high follow-up rate. However, long-term follow-up of a large number of ASCUS patients will better evaluate different detection methods and better manage ASCUS patients.

In our study, only 5 cases of CIN2+ occur among 103 women with ASCUS. The risk of developing a CIN2+ is six times higher in CIN1 than in cervical inflammation. Interestingly, the positive of all tests indicated the occurrence of CIN2+, which could be used to regulate the follow up after a negative colposcopy or CIN1 histology. From our study, we could also find that E6/E7 mRNA-positive is more serious than p16/Ki-67-positive and DNA-positive (risk ratio: 57.306 versus 3.629 and 23.842). Johansson et al. [22]. found that HPV E6/E7 mRNA positive patients have a higher risk of developing CIN2+ than the negative patients (OR 4.3, 95%CI 1.0–38.9) after conducting a 4.5-year follow-up of ASCUS patients. These dates supported that HPV E6/E7 mRNA assay may be a promisingly predictive method for detecting cervical precancer, and mRNA test should be performed on the specimens collected immediately before colposcopy. Paolo Giorgi Rossi’s result also support this view [23].

The positive rates of different tests were statistically different in different cervical lesions and the more serious the lesion, the higher the positive rates, which can be confirmed by many studies [24, 25]. HPV E6/E7 mRNA testing and p16/Ki-67 immunocytochemistry were identified to increase specificity for the detection of high-grade cervical disease compared to HPV DNA detection [26]. In women with HR-HPV-positive ASC-US and LSIL, sensitivity and specificity of p16/Ki-67 immunocytochemistry for detection of CIN3 were 90.6 and 48.6%, respectively [19]. A meta-analysis found that among ASCUS, sensitivity ranged from 0.64 to 0.92 (p16/Ki67 test) versus 0.91 to 0.97 (HPV DNA test); specificity ranged from 0.53 to 0.81 versus 0.26 to 0.44, respectively [27]. Our previous research suggested that HPV E6/E7 mRNA testing have higher specificity than HPV DNA test (40.30, 95%CI: 32.38–48.76 vs. 15.05, 95%CI: 10.27–22.74) [28].

Our study found that the specificity of p16/Ki-67 immunocytochemistry and E6/E7 mRNA testing for detection of CIN2+ was higher than HPV DNA test (*χ*^2^ = 26.277, *P* < 0.001, HPV DNA assay vs. HPV E6/E7 mRNA testing; *χ*^2^ = 80.707, *P* < 0.001, HPV DNA assay vs. p16/Ki-67 immunocytochemistry). The sensitivity between HPV DNA assay and HPV E6/E7 mRNA testing is no statistical difference (all sensitivities are 100, 95%CI: 60.97, 100), but statistical difference exists between HPV DNA assay vs. p16/Ki-67 immunocytochemistry (*χ*^2^ = 5.718, *P* = 0.023). This support that compared to HPV DNA assay, HPV E6/E7 mRNA testing improves the diagnostic specificity without reducing the sensitivity, while p16/Ki-67 immunocytochemistry improves the diagnostic specificity in a situation of reducing the sensitivity of diagnosis. However, Miriam Reuschenbach et al. [25] conducted a clinical experiment which compare the accuracy of CINtec p16INK4a-cytology, HPV E6/E7 mRNA and HPV DNA, they found that the specificity to detect high grade dysplasia was highest for CINtec p16INK4a-cytology (74.8% (65.5–82.3)), followed by HPV E6/E7 mRNA (71.2% (61.7–79.2)) and HPV DNA (63.4%(53.7–72.1)). Nicolas Wentzensen et al. [19] also found that p16/Ki-67 immunocytochemistry for detection of CIN2+ is close to HR-HPV DNA testing (*P* = 0.32), but a higher specificity(*P* < 0.0001). This difference may happen because the different of study method (our study is a prospective study, but other study is cross-sectional study).

The expression level of HPV E6/E7 mRNA was calculated in this study. The E6/E7 mRNA showed a higher expression levels in CIN2+ than in CIN1−, and the differences between the lesions were statistically significant (Z = − 3.407, *P* = 0.001). Thus, a ROC curve was used to further demonstrate the diagnostic performance of HPV E6/E7 mRNA testing, and the optimal cut-off value of mRNA testing is selected at 2097.09 copies/ml. Tong-Yu Liu’s and Ye-li Yao’s finding also supported that when an optimal cut-off value of mRNA testing was found out, the diagnosis accuracy will be improved [20, 29]. As the ROC shows, the AUC of p16/Ki-67 immunocytochemistry is the largest (AUC = 0.799, 95%CI: 0.649–0.908), followed by HPV E6/E7 mRNA testing and HPV DNA assay. Despite of it, p16/Ki-67 immunocytochemistry improves the diagnostic specificity while reducing the sensitivity of diagnosis. Thus, the question who is better in triaging ASCUS between p16/Ki-67 immunocytochemistry and HPV E6/E7 mRNA testing are unsolved. Regarding the opinions of our research group, HPV E6/E7 mRNA testing may be a promising and exact method in diagnosing CIN2+ among women with ASCUS considering the high sensitivity required for screening. p16/Ki-67 immunocytochemistry may also a wonderful tool, but more research about it in triaging ASCUS should be conducted to evaluate its diagnostic effect.

## Conclusion

The follow up after negative and CIN1 histology of ASCUS should be intensive for women with positive HPV E6/E7 mRNA, positive p16/Ki-67 immunocytochemistry and positive HPV DNA, and women with negative result of these test may be retested after longer intervals than currently performed. High expression of HPV E6/E7 mRNA could be a good candidate as a diagnostic biomarker to triage ASCUS superseding HPV DNA. p16/Ki-67 immunocytochemistry is suggested to be a good tool to triage ASCUS, but more research is needed to support this view.

## Data Availability

All data generated or analysed during this study are included in this published article.

## Abbreviations

ASCUS: Atypical squamous cells of undetermined significance
CIN1-: Cervical inflammation and CIN1 are referred as < CIN1-
ROC: Receiver operating characteristic
CIN1: Cervical inflammation or mild cervical intraepithelial neoplasia
CIN2 +: the diagnoses of CIN2, CIN3 and carcinoma are referred as ≥ CIN2
p16: p16INK4a
Pap: Papanicolaou
